# Characterizing masticatory motion of dogs using optical and electromagnetic motion tracking

**DOI:** 10.3389/fvets.2025.1625335

**Published:** 2025-07-03

**Authors:** Stephanie Goldschmidt, Hooi Pin Chew, Stephen Guy, Alex Fok

**Affiliations:** ^1^Department of Veterinary Clinical Sciences, University of Minnesota, St. Paul, MN, United States; ^2^Minnesota Dental Research Center for Biomaterials and Biomechanics, University of Minnesota, Minneapolis, MN, United States; ^3^Division of Operative Dentistry, Department of Restorative Sciences, University of Minnesota, Minneapolis, MN, United States; ^4^Department of Computer Science and Engineering, University of Minnesota, Minneapolis, MN, United States; ^5^Division of Biomaterials, Department of Restorative Sciences, University of Minnesota, Minneapolis, MN, United States

**Keywords:** chewing, masticatory motion, mastication, dogs, teeth

## Abstract

**Introduction:**

Accurate knowledge of masticatory motion across a variety of food materials is essential for *ex-vivo* testing and simulation of the food-teeth interaction. Yet, the masticatory motion has never been fully characterized in the domestic dog (*Canis lupus*), limiting our ability for *ex-vivo* modelling.

**Objective:**

The aim of this study was to characterize masticatory motion among a variety of different foods in beagle dogs using optical and electromagnetic motion tracking.

**Results:**

We confirmed that the masticatory pattern in the beagle is a hinge motion with no clinically meaningful horizontal motion of the mandible. The mouth opening was not significantly difference among different food and treat types regardless of food stiffness and force to fracture of the food, with a mean and standard deviation of 2.51  ±  0.33 (range 1.93–2.95) cm between the canine teeth during chewing. Conversely, frequency of chewing was influenced by food type, with kibbles having a significantly higher peak mean chewing frequency (2.93 Hz) compared to other feeds. Frequency of chewing was linearly correlated to the force to fracture of the food material (*p* = 0.03, R^2^ = 0.56), while stiffness of food did not significantly affect peak chewing frequency.

**Conclusion:**

Data from this study can guide ex-vivo modelling of the feed-teeth interaction for product design and testing, especially those that focus on prevention of periodontal disease and dentoalveolar trauma.

## 1 Introduction

Accurate knowledge of masticatory motion across a variety of food materials is essential for *ex-vivo* testing and accurate simulation of the food-teeth interaction. *Ex vivo* modelling directly informs diet/treat development and modification strategies, especially those that focus on prevention of periodontal disease or dentoalveolar trauma. Although masticatory motion has been characterized among herbivorous species (1–5), to the authors’ knowledge, chewing patterns have never been fully characterized in the domestic dog (*Canis lupus*).

In the dog, chewing primarily occurs between the carnassial teeth (maxillary fourth premolar and mandibular first molar) which interact in a vertical shearing motion. The maxillary first molar is also impactful as it is the only tooth with the ability to grind food when it interacts with the mandibular first molar (6). The chewing pattern is directly influenced by the motion of the temporomandibular joint (TMJ), which is a synovial joint comprised of the head of the mandible and the squamous portion of the temporal bone. In dogs it has been stated that the TMJ moves primarily in a vertical hinge-like motion with minimal translation (7, 8). Although horizontal motion was detected in 50% of cadaveric dog specimens with approximately 2 mm of movement in the lateral direction (7), this limited horizontal motion is likely clinically negligible. Further, it has been theorized that any lateral mandibular motion in dogs actually occurs due to opening of the mandibular symphysis that directly contributed to lateral sliding of the condyles rather than horizontal movement confined within the TMJ (9). However, these findings have not been confirmed *in vivo*. To the authors’ knowledge, there is only limited data on masticatory motion in dogs, with one paper evaluating chewing rate among different breeds (10). There is also no data to inform how motion changes with different food materials. Lack of accurate data on the masticatory motion of the domestic dog directly limits our ability for *ex-vivo* modelling of the tooth-food interaction. The aim of this study was to characterize masticatory motion among a variety of different foods in beagle dogs using optical and electromagnetic motion tracking.

## 2 Materials and methods

### 2.1 Motion analysis

Six 1-year-old healthy beagle dogs (2 male, 4 female) from a commercial breeding colony had their chewing patterns evaluated among a variety of different food materials. The study was approved by the University of Minnesota Institutional Animal Care and Use Committee (#2003-37962A). All dogs had normal occlusion and no previous oral trauma or extractions. All dogs were housed together in university facilities during the length of the trial and were observed during all morning feedings of maintenance kibble and treats. Masticatory motion data was not collected during evening feedings of the maintenance diet, but the amount of food eaten was recorded.

**Figure 1:**
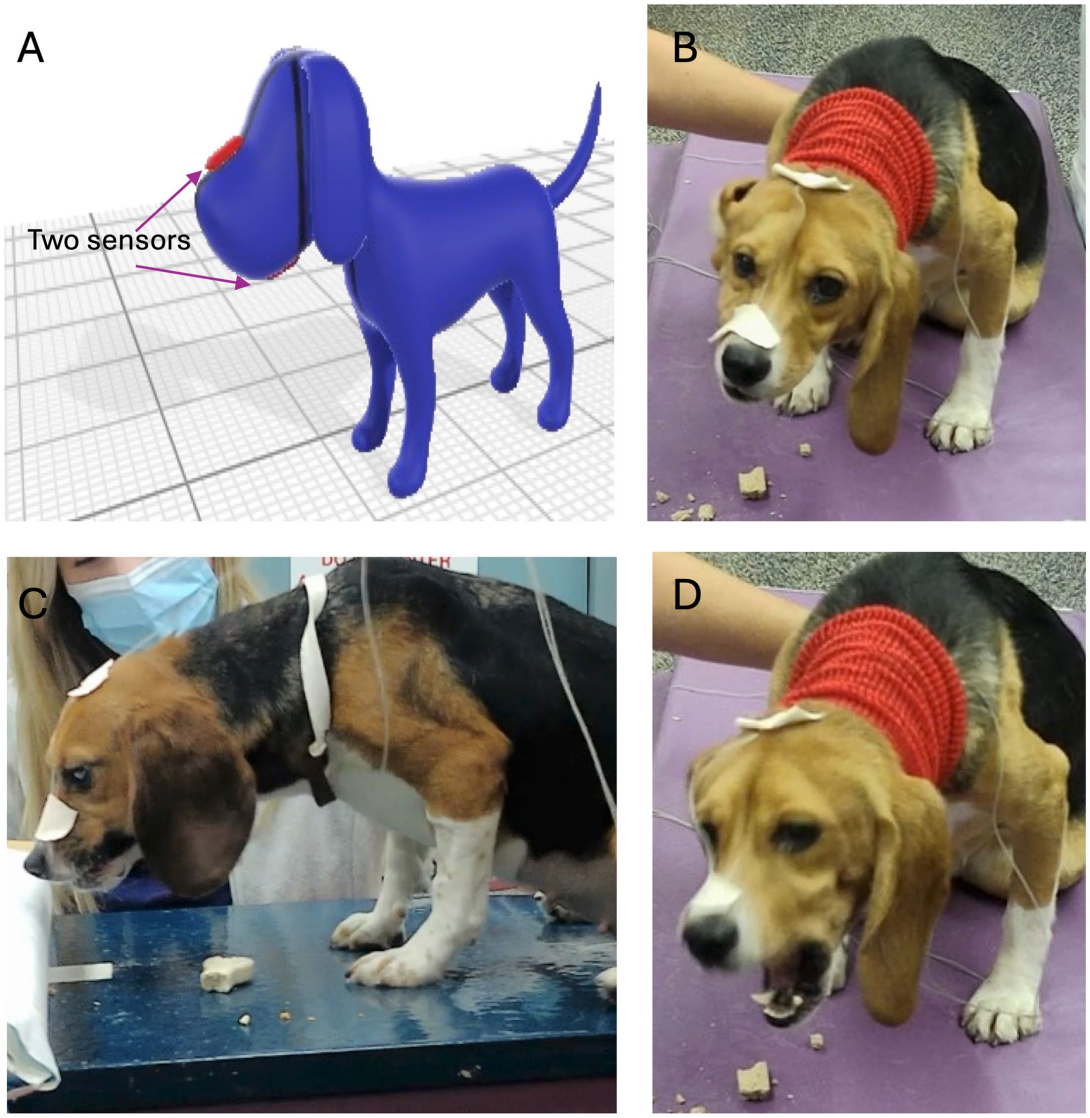
Microsensors used during chewing evaluation. **(A)** Sensors were placed at the level of the maxillary and mandibular canines and secured with tape. **(B–D)** Dogs were videotaped during eating with 2 cameras position 90 degrees from one another.

Masticatory motion was captured with two electromagnetic 1.8 mm microsensors (Micro Sensor 1.8, Polhemus, Colchester, VT). The nominal accuracy of the sensors for X, Y, Z position was 0.15 cm and 0.40 ° for sensor orientation. The sensors were attached to the skin at the level of the maxillary and mandibular canine teeth (Figure 1). They allowed for real-time motion tracking at 60 Hz for all 6 degrees of freedom. Each dog was fed a maintenance dry dog food (Kibble; Blue Buffalo, Blue Life Protection Formula Adult Chicken and Brown Rice Recipe, Minneapolis, MN), throughout the trial. For each dog, data was collected for the maintenance kibble a minimum of 20 times, giving a total of 144 data sets from the 6 dogs. All dogs readily ingested the maintenance kibble. Following feeding of the maintenance diet, all dogs were offered eleven different treats of various textures and sizes (Table 1; Supplementary Figure 1). No dog was forced to ingest any alternative food or treat, thus there was varied sample sizes on alternative food material (not kibble), pending the dog’s interest.

**Table 1:** Food material fed to the beagle dogs for masticatory motion analysis and mechanical testing.

Brand name of product	Short name of product for study	Force to fracture (N) mean (SD)	Stiffness (N/mm) mean (SD)	Dimesions (cm)
OraVet Dental Hygiene Chews for Dogs	OraVet	665.72 (101.86)	31.16 (4.13)	6.99 × 0.94 × 1.42
Checkups Chews for Dogs	Checkup	1081.69 (228.27)	84.42 (11.92)	12.1 × 3.81 × 1.27
Minties dental treats	Minties	709.37 (268.55)	70.99 (12.75)	8.26 × 2.54 × 1.27
Member’ Mark Dental Treats	Members mark	863.92 (142.13)	73.16 (12.34)	12.7 × 3.4 × 1.4
Prescription Diet Canine a/d	Canned food	Not available	Not available	Not available
Canine Greenies	Greenies	381.91 (64.5)	42.93 (19.52)	10.49 × 2.49 × 1.19
Milk bone original biscuits medium dog treats	Milkbone	81.29 (17.17)	38.33 (8.19)	6.99 × 2.90× 1.30
Prescription Diet Canine t/d: Original Bites	TD	220.78 (68.26)	29.89 (5.03)	2.54 × 2.54 × 2.54
C.E.T. VEGGIEDENT Fresh Chews for Dogs	Veggiedent	790.39 (269.8)	287.81 (50.66)	9.5 × 2.54 × 1.52
Dentalife daily oral care	Dentalife	349.78 (51.62)	43.35 (6.54)	10.16 × 1.9 × 1.9
Pedigree Dentastix Advanced	Dentastix	408.3 (86.88)	125.89 (44.5)	8.9 × 1.65 × 1.29
CET enzymatic oral hygiene* chews for dogs*	CET rawhide	1056.27 (201.26)	129.57 (51.61)	Variable
Whimzees dental treats*	Whimzees	1157.74 (470.4)	304.35 (117.03)	10.5 × 3.6× 2 cm
Tartar Shield soft rawhide * chews*	Tartar shield rawhide	480.71 (201.06)	28.64 (13)	20.32 × 13.21 × 2.54
Purina DH dental diet*	DH*	100.66 (20.97)	39.35 (6.86)	2.8 × 2.8 × 2.8
Blue Buffalo, Blue Life Protection Formula Adult Chicken and Brown Rice Recipe	Kibbles	Not available	Not available	1.06 × 1.26 × 0.44

*These were only included in mechanical testing and were not ingested by the beagles.

**Figure 2:**
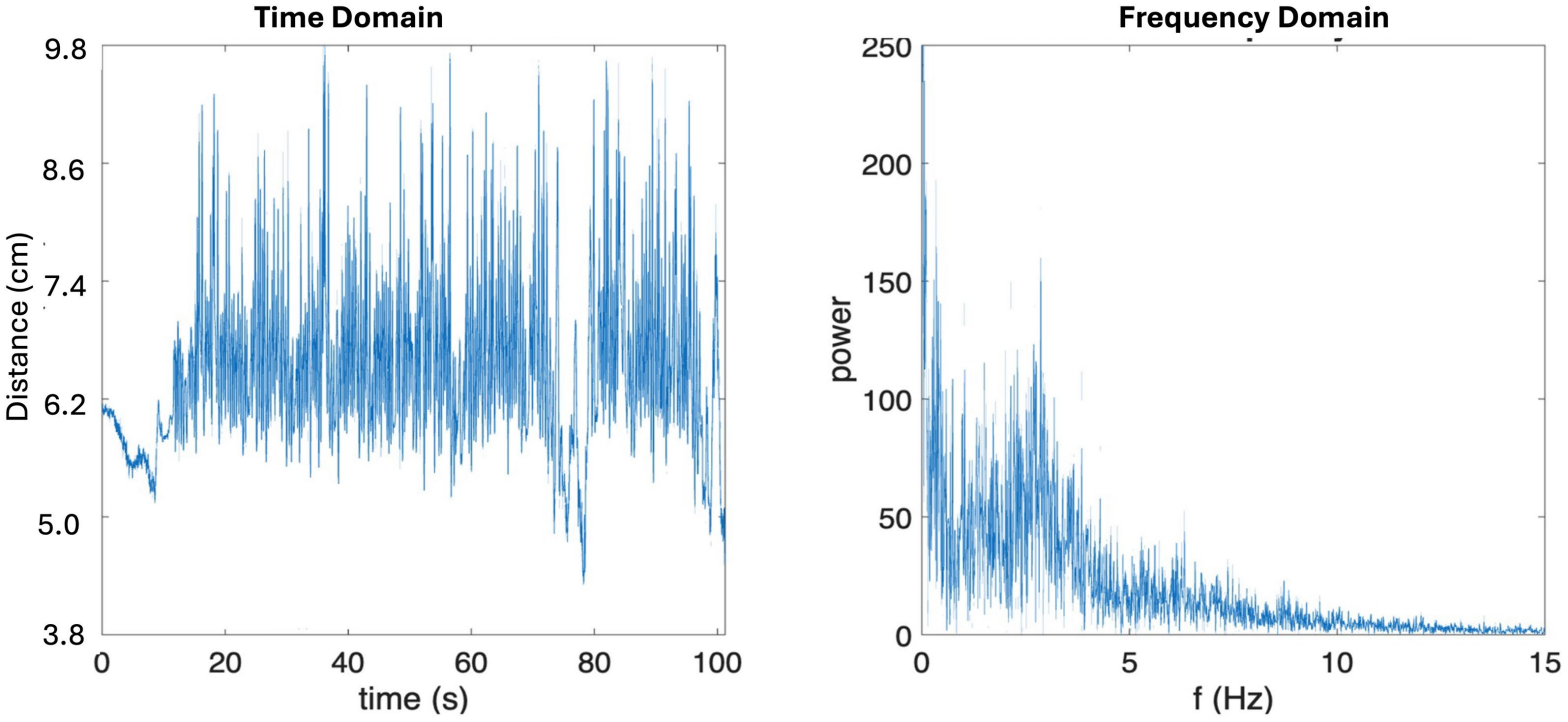
Sample microsensor data in time domain (left) and frequency domain (right). Note in the time domain sample data, there is a 20 s interval of non-chewing behavior prior to chewing motion where you can see the effect of head movement on raw data.

Maximum mouth opening was calculated by evaluating the motion of the mandibles relative to the maxilla. Specifically, by calculating the distance between the 2 motion tracking sensors using the equation Sqrt[(x2-x1)^2^ + (y2-y1)^2^ + (z2-z1)^2^], where x1, y1 and z1 are the coordinates of Sensor 1 in the Cartesian coordinate system, and x2, y2 and z2 are those of Sensor 2. Chewing frequency was derived by transforming the raw temporal data of mouth opening into the frequency domain using Fast Fourier Transform (FFT; Figure 2).

Repeatability of the device was evaluated by looking at frequency of chewing during maintenance diet ingestions. All beagles were fed ¾ cup of the maintenance kibble on a minimum of 20 occasions with an approximately 24-h rest interval between sessions. During each session, the device recorded the frequency of mouth opening for a minimum of 2 min of chewing.

In addition to the microsensors, two video cameras (1920 × 1,080 pixels, Sony, New York, NY) were also placed parallel and perpendicular to the dog at approximately 90-degree relative angle to each other to capture both frontal and sagittal views of chewing. All feedings were recorded. Videos were reviewed as needed during data review to correct/explain aberrant head motion during chewing that needed to be corrected for data analysis.

Video footage was also utilized to evaluate how consistent the observed chewing motion was with a single-hinge or “unidimensional” vertical chewing model. The videos were synchronized temporally, and 33 feature correspondences between two sample frames were manually identified to establish the fundamental matrix, linear camera intrinsics, barrel lens distortion, and the relative camera positions (up to an arbitrary scale factor). During a 12-s clip of the dog chewing, 4 key points were manually tracked in each frame: the dog’s right maxillary canine tooth, nose, and two forehead points marked by tracking tape. 3D positions for each of these points were established using non-linear triangulation via reprojection error minimization. Finally, the chewing motion was rotationally rectified with the largest axis of motion aligned along the vertical/y-axis representing the primary dimension of chewing motion.

### 2.2 Relationship between chewing frequency and mouth opening with the mechanical properties of the feed material

**Figure 3:**
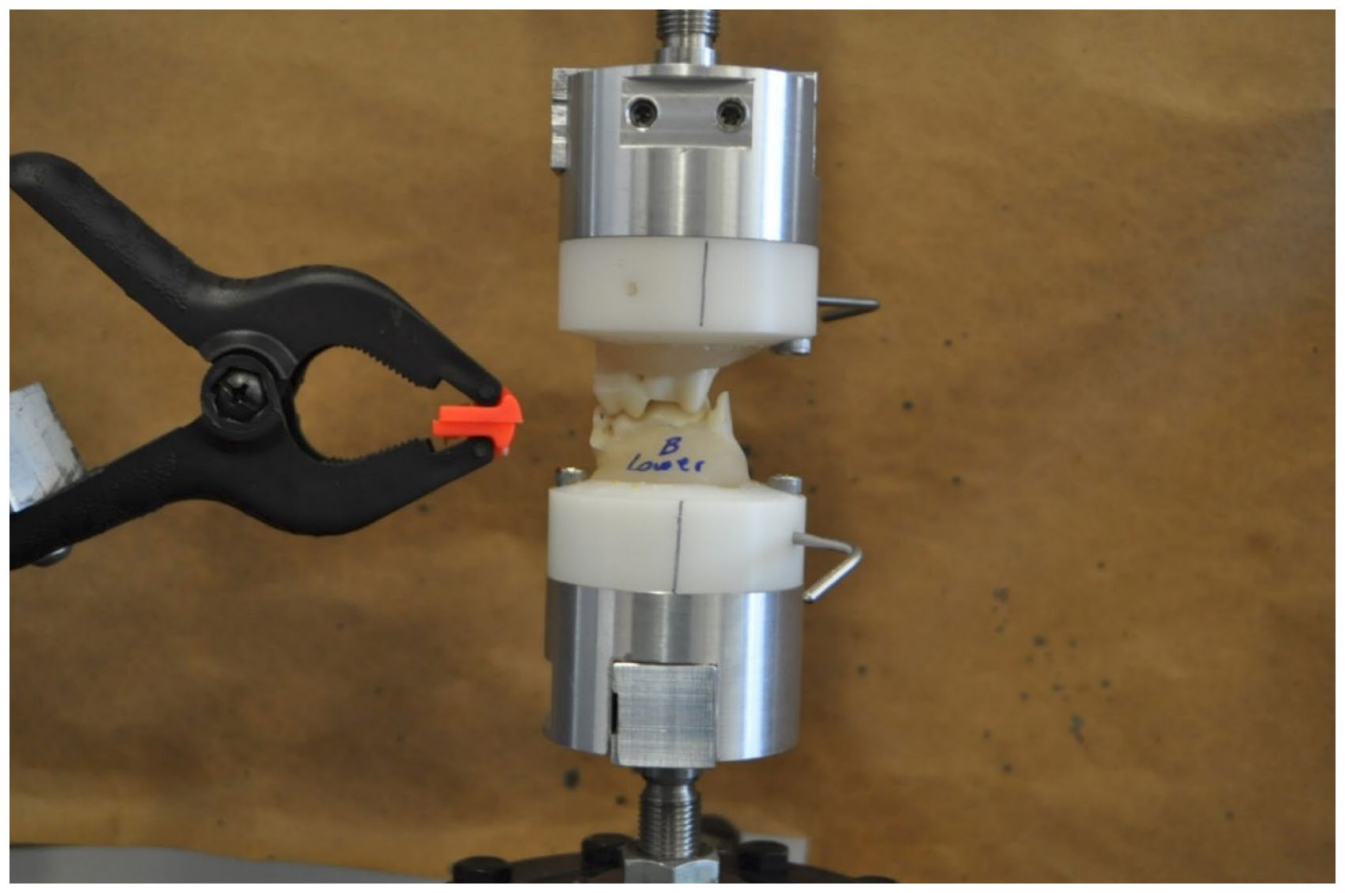
Zirconium maxillary fourth premolar and first molar teeth mounted to a load cell for mechanical testing of different food materials. The pliers in the image are for holding the treat during testing.

To explore the possible relationship between masticatory patterns and mechanical properties of food, biomechanical testing of food was performed. Impressions (Vinyl polysiloxane, Henry Schein, Melville, NY) of the maxillary fourth premolar and first molar as well as the mandibular first molar were obtained from a cadaveric beagle dog that was euthanized for reasons unrelated to the study. The impressions were utilized to create custom zirconia teeth for biomechanical testing. The zirconia teeth were anatomically aligned and mounted with orthodontic resin into Teflon rings. The rings were then mounted to a tabletop servohydraulic universal testing machine (858 Mini Bionix II, MTS, Eden Prairie, MN) (Figure 3). Each food/treat was loaded at a displacement speed of 0.5 mm/s to a limit force of 3,500 N. The force to fracture was identified from a rapid decrease of force of >50% on the force–deflection curve. Stiffness was calculated from the slope of the force–deflection curve. Each food/treat sample was tested five times, from which the mean fracture force and stiffness were calculated.

### 2.3 Statistical analysis

Mean chewing frequency (F) and mouth opening (O) were calculated for each food type. Statistical analysis was performed in SPSS v29 (IBM) and significance was set at *p* < 0.05. The Kolmogorov–Smirnov test was used to determine the distribution of these two variables, and it was found that both were normally distributed (*p* > 0.05). Therefore, differences of the mean chewing frequency and mouth opening between feed materials were compared using the ANOVA test, followed by the Tukey *post hoc* test. The Pearson Correlation test was performed to assess the association between mouth opening and peak frequency and the mechanical properties of the food, i.e., stiffness and force to fracture. The association between mouth opening and peak frequency with the thickness of the food material was computed but was not assessed statistically due to insufficient range of data points.

## 3 Results

**Figure 4:**
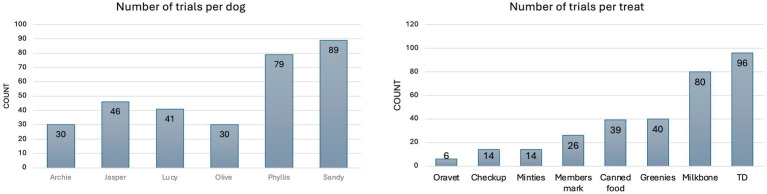
Total number of alternative food/treats ingested per dog (left) and total number of each alternative food/treats ingested throughout the entire trial (right).

Four hundred and seventy-three measurements were performed in the 6 dogs, with varying trials per type of food/treat per dog (Figure 4), as the dogs had varied interest in different alternative feed types other than the maintenance diet. There was minimal variation in chewing frequency and mouth opening between the 6 dogs (Supplementary Figure 2). The within-subject standard deviation for chewing frequency was 0.27 Hz and the coefficient of variation was 9.18%, reflecting low relative variability and high repeatability of the device for chewing measurement.

**Figure 5:**
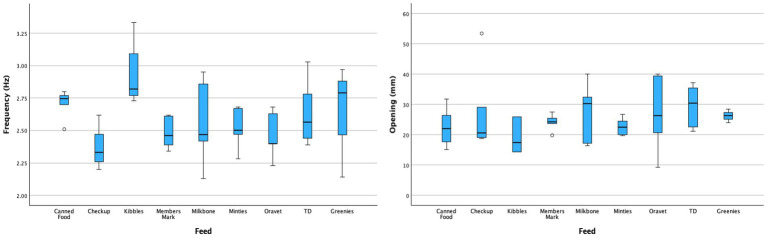
Box and whisker plots of chewing frequency (left) and mouth opening (right) per food type.

For comparison of the mean mouth opening and chewing frequency amongst the types of food, Veggiedent, Dentalife and Dentastix were excluded from statistical comparison due to insufficient data points. Mean mouth opening ranged from 1.93 to 2.95 cm for the different food types but was not significantly different among them (*p* = 0.47). Conversely, mean chewing frequency ranged from 2.37 to 2.93 Hz for the different food types and was significantly higher for kibble (*p* < 0.05) compared to most other food types except for canned food (*p* = 0.59), TD (*p* = 0.21), and Greenies (*p* = 0.19) (Figure 5). There was no significant correlation (*p* = 0.142, R^2^ = 0.32) between mouth opening and peak chewing frequency regardless of food type.

Based on video analysis, the separation of the maxilla and mandible seen during chewing was in a 3.34:1 ratio between vertical and horizontal directions with more horizontal motion towards the maximum closing rather than maximum opening. That is, 77% of the range of lower jaw motion during maximum opening was contained entirely within the primary vertical dimension. This is consistent with a single-hinge chewing model to within the accuracy of the camera-based tracking techniques used.

### 3.1 Relationship between chewing frequency and mouth opening with the mechanical properties of the feed material

**Figure 6:**
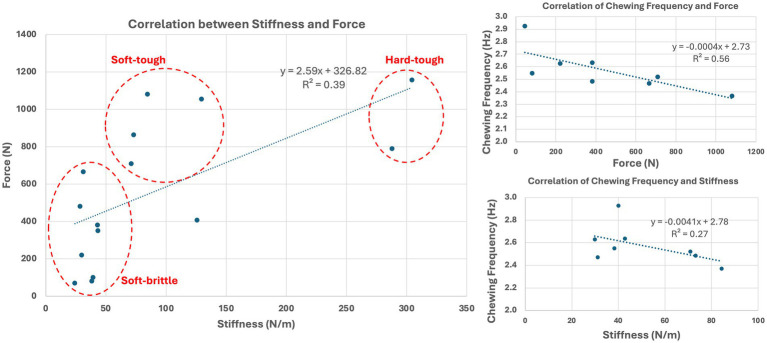
Correlation between food stiffness and strength (left) showing that the feed materials could be broadly categorized as soft-brittle, soft-tough, or hard-tough. Correlation between the frequency of chewing with force to fracture and stiffness of the food materials (right).

Food stiffness and force to fracture were highly varied among the tested feed materials (*n* = 14) and ranged from 24 to 304 N/mm and 70 to 1,158 N, respectively (Table 1). Based on their strength and stiffness characteristics (Figure 6), the feed materials could be broadly categorized as soft-brittle, soft-tough, or hard-tough. The fracture force and stiffness relationship may be related with a fractional power curve, but at low stiffness a straight line suffices and significant linear correlation between food stiffness and force to fracture is observed (*p* = 0.013, R^2^ = 0.39).

Chewing frequency was significantly correlated with force to fracture (*p* = 0.03, R^2^ = 0.56) but not significantly correlated with stiffness (*p* = 0.183, R^2^ = 0.27). Although there was no significant correlation with stiffness, it is noted that there is similar correlation trend to that of force to fracture (Figure 6). Conversely, mouth opening was not significantly associated with stiffness nor with force to fracture (*p* > 0.05).

The thickness of most of the treats offered and routinely ingested by the beagles was approximately 1.25 cm. There was weak positive linear correlation between the thickness of the diet/treat and mouth opening (R^2^ = 0.36) but poor correlation with chewing frequency (R^2^ = 0.18).

## 4 Discussion

This is the first study to characterize the masticatory motion of the beagle dog. This study confirmed that dogs chew primarily in a hinge motion with minimal horizontal jaw movement. It was found that despite highly varied mechanical properties of the food material, there was no statistically significant difference in the mouth opening among food types. Frequency of chewing, on the other hand, was significantly different for kibble compared to other food/treat types and appeared to be driven primarily by food strength (force to fracture) rather than stiffness.

This is also the first study to describe the mechanical properties of commonly prescribed treats and food. Most treats were chosen as they are marketed for periodontal prevention and have received Veterinary Oral Health Council (VOHC) approval (11). Of note, 4/14 tested food materials had a force to fracture that was higher than what has been reported to result in fracture of the maxillary fourth premolar based on cadaveric biomechanical studies. Previous work has revealed fracture occurs above a force of 831 N and 1,281 N for root canaled and intact fourth premolar teeth, respectively (12, 13). It is unknown if this force would be truly generated during clinical chewing to crush a treat. Limited and contradictory literature currently exists on the *in vivo* bite force in dogs, and most is centered on dogs that are excited (biting a sleeve or very high value treat) or anesthetized with the muscles of mastication stimulated by electrodes, thus not properly representing the clinical situation (14, 15). A recent study used a biomechanical model to explore the relationship between skull shape and bite force and reported a wide range of maximal bite forces from 124–1,380 N for the canines and 229–2,364 N for the carnassial teeth (8). Likely the daily chewing force is much lower than the maximal bite force, as is described in humans (16). Indeed, repeated biting with a moderate force at different locations of a food is used by all animals to break down hard foods. Accordingly, most *ex-vivo* biomechanical studies in dogs use a bite force of approximately 200 N for cyclic loading (17). Furthermore, the force to fracture may be rate dependent, with the fracture force decreasing with increasing rate of loading. We elected to load with a slow speed to ensure that the stiffness (store modulus) could be measured independently from the rate dependent viscous component (loss modulus), which is standard for initial stiffness and strength testing of an unfamiliar food. Additional testing with a faster loading speed more comparable to chewing rates in dogs and historical biomechanical fracture studies (12, 13) would have also been advantageous. Despite these limitations, it does bring up the point that biomechanical testing should be a factor in food development to avoid potential tooth fracture. No dentoalveolar trauma was noted in our cohort of dogs, but the stiffest/strongest treats also had some of the lowest sample sizes and were not routinely ingested, limiting the ability to make clinical conclusions.

We confirmed that at a minimum of 77% of the time the chewing pattern in dogs is a hinge motion, confirming the cadaveric work by Lin et al. (7). This justifies the use of a uniaxial test in this study to measure the mechanical properties of the foods tested. Although, there was presence of horizontal motion (peak of 0.75 cm) noted on video analysis, the horizontal contribution to the total maximum separation between the jaws was small making a hinge motion the more fitting approximation of chewing pattern. We cannot confirm that the hinge motion occurs 100% of the time due to distinct limitations in methodology. Our original aim was to evaluate hinge versus lateral motions with the microsensors, yet there was too much additional noise in the data secondary to head motion during eating to accurately extrapolate this data. Thus, video analysis was utilized to further analyze the motions. Blurry motion in the videos and certain videos without perfect alignment means that some of the data had to be discarded as it could not be calibrated between recordings from the two cameras (parallel and perpendicular). Future work using videos to analyze chewing motion would benefit from the use of 3 high-resolution cameras, rather than 2 as well as the addition of a tracking tool that would allow accurate capture of lateral movement both at the TMJ and the mandibular symphysis.

Fast Fourier Transform (FFT) is an efficient algorithm for extracting the frequencies and their amplitudes from a discrete sequence of values in space or time (18). FFT of the microsensor data revealed that the mouth opening did not differ significantly with food types, although this may be driven by the high standard deviation in the data set. Conversely, frequency was significantly higher for kibble compared to the majority of other feed materials except a dental diet (food 8) and a soft dental chew (food 6). Force to fracture appeared to be the primary driver, with treats requiring less force to fracture having a significantly higher frequency of chewing. For modelling, we recommend that treats with a force to fracture under 1,000 N be chewed at a higher frequency (2.8 Hz compared to 2.5 Hz). This value was chosen based on the force at which the relationship between stiffness and force to fracture became non-linear.

Similar work in humans shares some similarities to our canine population including that food strength is not associated with mouth opening (18). Conversely, food hardness has been shown to directly affects incisor gape (mouth opening) and velocity of chewing in humans (19–21). In the beagles, food strength, but not stiffness, was significantly associated with chewing frequency. Of note, despite the lack of statistically significant correlation between chewing frequency and stiffness, the trend was similar to that of force to fracture and frequency, and the stiffness and force to fracture of the foods were linearly correlated. This suggests that stiffness also plays a role in chewing frequency in dogs, similar to humans. Further, impactful findings correlating biomechanic properties, including food size, with mouth opening may have been masked by the large standard deviation of mean mouth opening coupled with the relatively uniform thickness of diets/treats evaluated.

Further, although two dogs did have a consistently higher chewing frequency than others, in numerical terms this change in frequency was very small. The minimal inter-dog variation may be because dogs were all from the same breeding colony and share distinct similarities due to their shared lineage. It may also be that that similar head shape and size across one breed (e.g., beagles) is more related to chewing pattern than the food mechanical properties. That being said, minimal variation in masticatory motion may potentially be applicable to all domestic dogs regardless of breed (and body mass) as has been found with stable chewing rates among different sized domestic dogs (10). This is believed to be due to loss of evolutionary pressure in domesticated animals to change their chewing patterns across different body masses to meet nutritional and biologic needs as they are fed commercial diets by humans (10, 15). Furthermore, there also must be behavioral consideration given to the techniques used within this study, which offered food and treats in an unnatural environment where dogs were fed alone and while being directly monitored/recorded, which may have impacted the masticatory motion and willingness to ingest certain treats (15). Further work looking at the masticatory motion among different sized dogs with varied head shape as well as in groups compared to individual feeding would be of interest. Beagles were chosen as these are a very commonly utilized research breed, thus data from this breed would be directly applicable to many *in vivo* research studies that have been already performed.

The primary limitations of this study include the small sample size for some treats that were of low interest and the use of only one breed. Further, treats thickness were compared to mouth opening and frequency as this was the most uniform direction a treat was ingested/introduced to the carnassial teeth, but a dog may choose clinically to introduce the treat from a different direction (making width or length more appropriate). The microsensors utilized within the study group also have distinct limitations including the introduction of small errors due to skin movement and increased noise due to head movement during eating leading to the inability to confirm the true vertical and lateral movements of the mandibles. It should also be noted that the mouth opening as measured between the microsensors is not a true gape angle and will differ in different sized patients. To remedy this in dogs of different breeds, the length of the mandible should be measured to scale the mouth opening at the carnassial teeth accordingly. Further, although repeated testing of kibble in each dog showed minimal variation, to confirm repeatability of use of microsensors as part of the methodology, testing on an *ex-vivo* model first would have been ideal. Last, chewing force could not be obtained *in vivo* as this would have changed the chewing pattern of the dogs (require a sensor on the food), but would be of high interest in the future for *ex-vivo* modelling.

## 5 Conclusion

This study found that the mean chewing frequency and mouth opening are 2.59 Hz and 2.5 cm, respectively, in beagle dogs chewing a variety of food material. Mouth opening is mainly hinge like and is not significantly affected by mechanical properties of food, while chewing frequency decreases with force to fracture of feed materials. These guidelines can be utilized for further work testing the food-tooth interaction.

## Data Availability

The raw data supporting the conclusions of this article will be made available by the authors, without undue reservation.
