# Supplementary figures and images for: Characterizing masticatory motion of dogs using optical and electromagnetic motion tracking
Source: Front Vet Sci. 2025 Jul 3;12:1625335. doi: 10.3389/fvets.2025.1625335 (PMC12268705; doi:10.3389/fvets.2025.1625335)

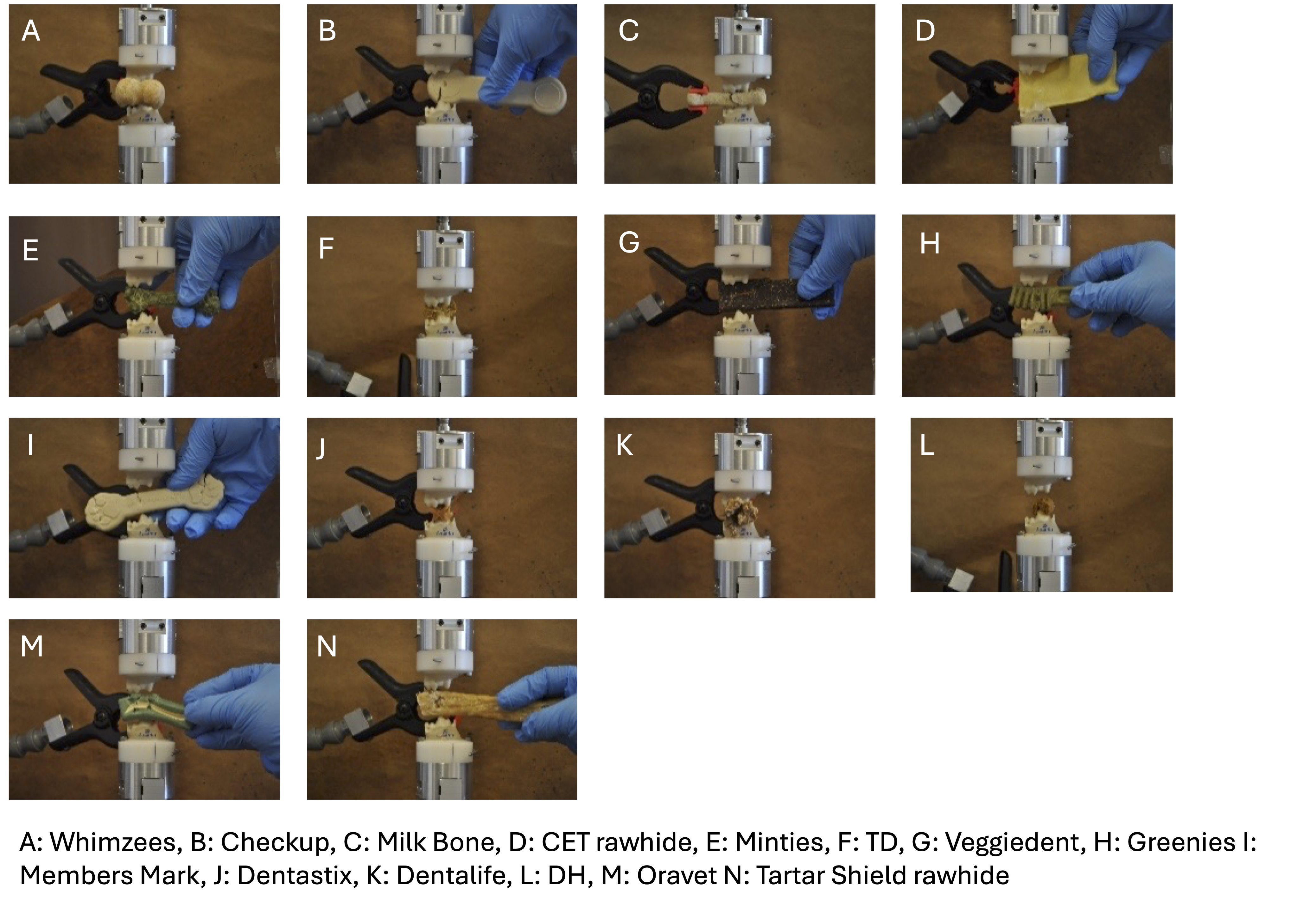

Supplement: Supplementary file 1 [file Image_1.tiff]

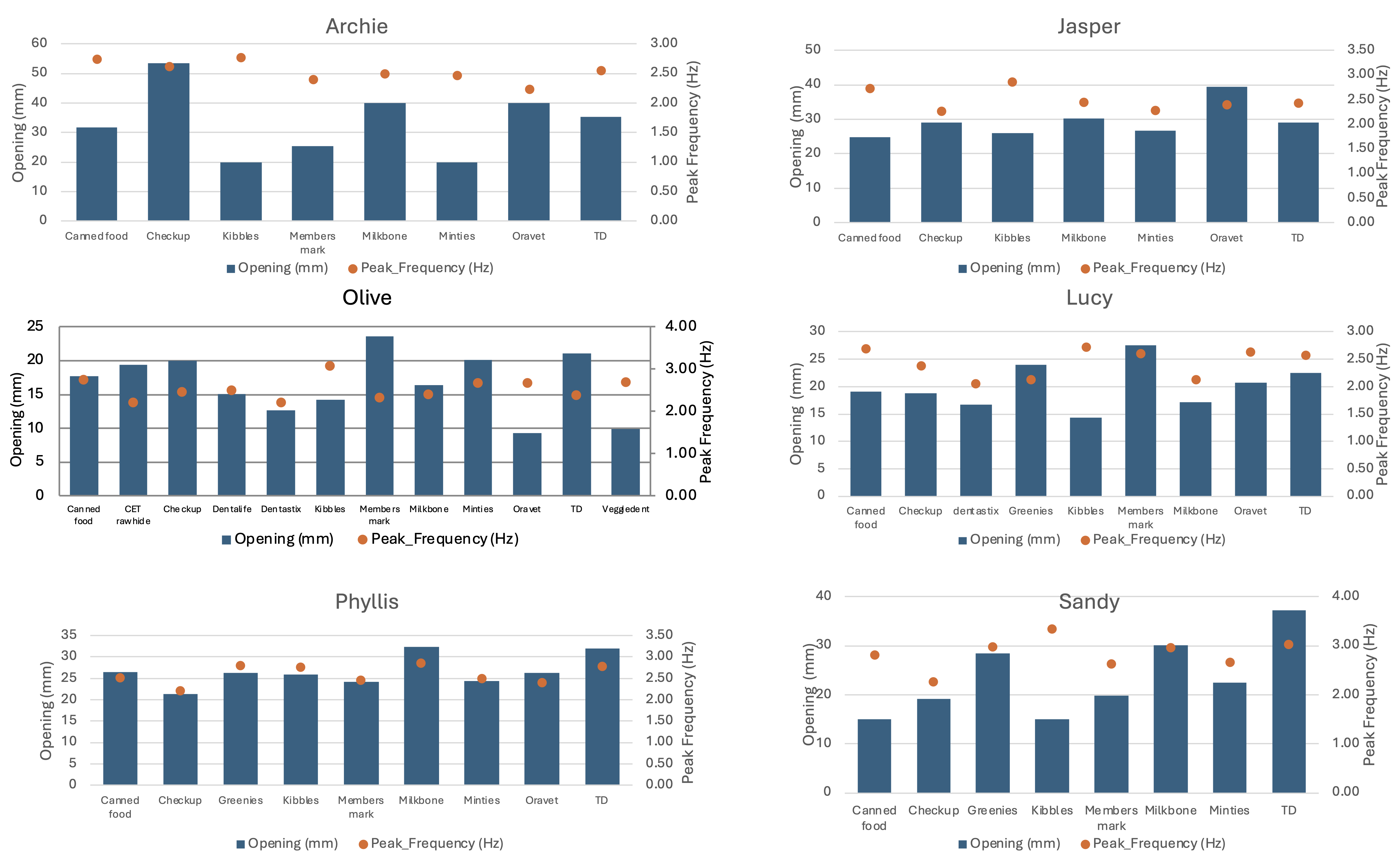

Supplement: Supplementary file 2 [file Image_2.tiff]
